# Formation Conditions and Sedimentary Characteristics of a Triassic Shallow Water Braided Delta in the Yanchang Formation, Southwest Ordos Basin, China

**DOI:** 10.1371/journal.pone.0119704

**Published:** 2015-06-15

**Authors:** Ziliang Liu, Fang Shen, Xiaomin Zhu, Fengjie Li, Mengqi Tan

**Affiliations:** 1 State Key Laboratory of Oil and Gas Reservoir Geology and Exploitation, Chengdu University of Technology, Chengdu, China; 2 School of Energy, Chengdu University of Technology, Chengdu, China; 3 State Key Laboratory of Geological Processes and Mineral Resources, China, University of Petroleum, Beijing, China; 4 Institute of Sedimentary Geology, Chengdu University Technology, Chengdu, China; Centro de Investigacion Cientifica y Educacion Superior de Ensenada, MEXICO

## Abstract

A large, shallow braided river delta sedimentary system developed in the Yanchang Formation during the Triassic in the southwest of the Ordos basin. In this braided delta system, abundant oil and gas resources have been observed, and the area is a hotspot for oil and gas resource exploration. Through extensive field work on outcrops and cores and analyses of geophysical data, it was determined that developments in the Late Triassic produced favorable geological conditions for the development of shallow water braided river deltas. Such conditions included a large basin, flat terrain, and wide and shallow water areas; wet and dry cyclical climate changes; ancient water turbulence; dramatic depth cycle changes; ancient uplift development; strong weathering of parent rock; and abundant supply. The shallow water braided river delta showed grain sediment granularity, plastic debris, and sediment with mature composition and structure that reflected the strong hydrodynamic environment of large tabular cross-bedding, wedge cross-bedding, and multiple positive rhythms superimposed to form a thick sand body layer. The branch river bifurcation developed underwater, and the thickness of the sand body increased further, indicating that the slope was slow and located in shallow water. The seismic responses of the braided river delta reflected strong shallow water performance, indicated by a progradation seismic reflection phase axis that was relatively flat; in addition, the seismic reflection amplitude was strong and continuous with a low angle and extended over considerable distances (up to 50 km). The sedimentary center was close to the provenance, the width of the river was large, and a shallow sedimentary structure and a sedimentary rhythm were developed. The development of the delta was primarily controlled by tectonic activity and changes in the lake level; as a result, the river delta sedimentary system eventually presented a “small plain, big front” character.

## Introduction

The concept of a shallow water delta was first introduced by Fisk. In Fisk’s research on the Mississippi River Delta, he divided the fluvial-dominated delta into two types: deep and shallow water[1]. Donaldson further summarized the concept of a shallow water delta and discussed the effects of water depth on delta development[2]. Postma divided the low-position fluvial-dominated deltas of basins into shallow and deep water types and further distinguished 8 types of shallow-water deltas[3]. Based on the classification of Postma, the study of shallow water deltas has gradually progressed in recent years, mainly with respect to delta formation dynamics, sedimentation processes, microfacies recognition and sand body configuration[4,5,6,7,8,9]. Such studies have proposed that distributary channel sand form the skeleton of shallow water deltas, with the mouth bar undeveloped at the front of shallow water deltas. The sand body distribution is significantly affected, and it is not only distributed in a strip but also concentrated in certain areas.

In the 1980s, Chinese scholars began to study shallow water deltas. Gong Shaoli discussed a mainly fluvial shallow water delta that had developed in Henan Yu County in the late Early Permian[10]. Su Chunqian observed distinctly different sediment types between the southern margin of the Ordos basin carboniferous delta and the traditional Gilbert delta. These deltas’ sedimentary sequence was given priority and thinned going upward[11]in the presence of the delta’s front forward coarsening upward sequence, which has the distinct characteristics of a shallow water delta. Li et al. studied structures of the Carboniferous and Permian periods and discovered an enormous complex shallow water delta sedimentary system in which a distributary channel was developed along with frequent branches with a small shunt that were accompanied with limited front delta deposition[12]. Lou et al. indicated that a shallow water delta had developed in the Putaohua oil layer in the northern Songliao basin; the delta had the unique characteristic of being an underwater distributary channel and had developed by the spread of delta-front deposits. A delta plain and delta front developed, the former developing into a flat structure and the latter spreading over the plain. There was no Gilbert-type mode of delta formation with a top set, foreset, and bottom set[13].

In the 21st century, the study of shallow water deltas by Chinese scholars has mainly focused on the Songliao basin[14,15,16,17,18,19,20,21,22,23,24], Ordos basin[25,26,27,28,29], and Sichuan basin[30]. The study of shallow water deltas has revealed their outstanding characteristics, with underwater distributary channels as the main body and not debouch bar sediments, unlike conventional Gilbert deltas, which show small plain, big front characteristics and present a broad distribution over plains. The Ordos basin shows the characteristics of a basin full of sand. Furthermore, fluvial and paleotopographic lake-level changes affected the growth of shallow water deltas. Shallow water deltas developed on a flat terrain due to the structural stability of the platform and the presence of shallow water, under the formation conditions of a subsidence over an overall slow basin depression.

The delta located at the margins of the southwest Ordos basin of the Yanchang Formation has always been a hotspot for geologists in China and abroad, especially in recent years. An oilfield with reserves of 100 million tons was found close to the Xifeng, Huanxian, Zhenbei, HuaQing areas, promoting additional research on depositional systems, source directions, sequence filling style and lithofacies paleogeographic distribution. Certain researchers believe that the research areas were mainly underwater alluvial fans of deposition[31], whereas others have advanced the viewpoint that the region was an ordinary river delta[32] as well as a theory involving fan delta deposits[33]. More recently, the formation of braided river deltas and sandy debris flows has been proposed[34]. Therefore, to strengthen the study of sedimentary system types and sedimentary characteristics, it is more advantageous to conduct precise research on oil and gas exploration and development in the future. Based on field work conducted in the Yan River and five additional outcrop sections and a detailed description of the cores of more than 70 wells, it was proposed that the southwest margin of the Ordos basin was full of large areas of shallow water that persisted across the gentle slope topography. In addition, there were frequent elevations of lake levels that resulted in a distinctive shallow braided river delta sedimentary system during the Triassic. In this paper, the sedimentary characteristics of these areas are discussed.

## Regional Geological Background

**Fig 1:**
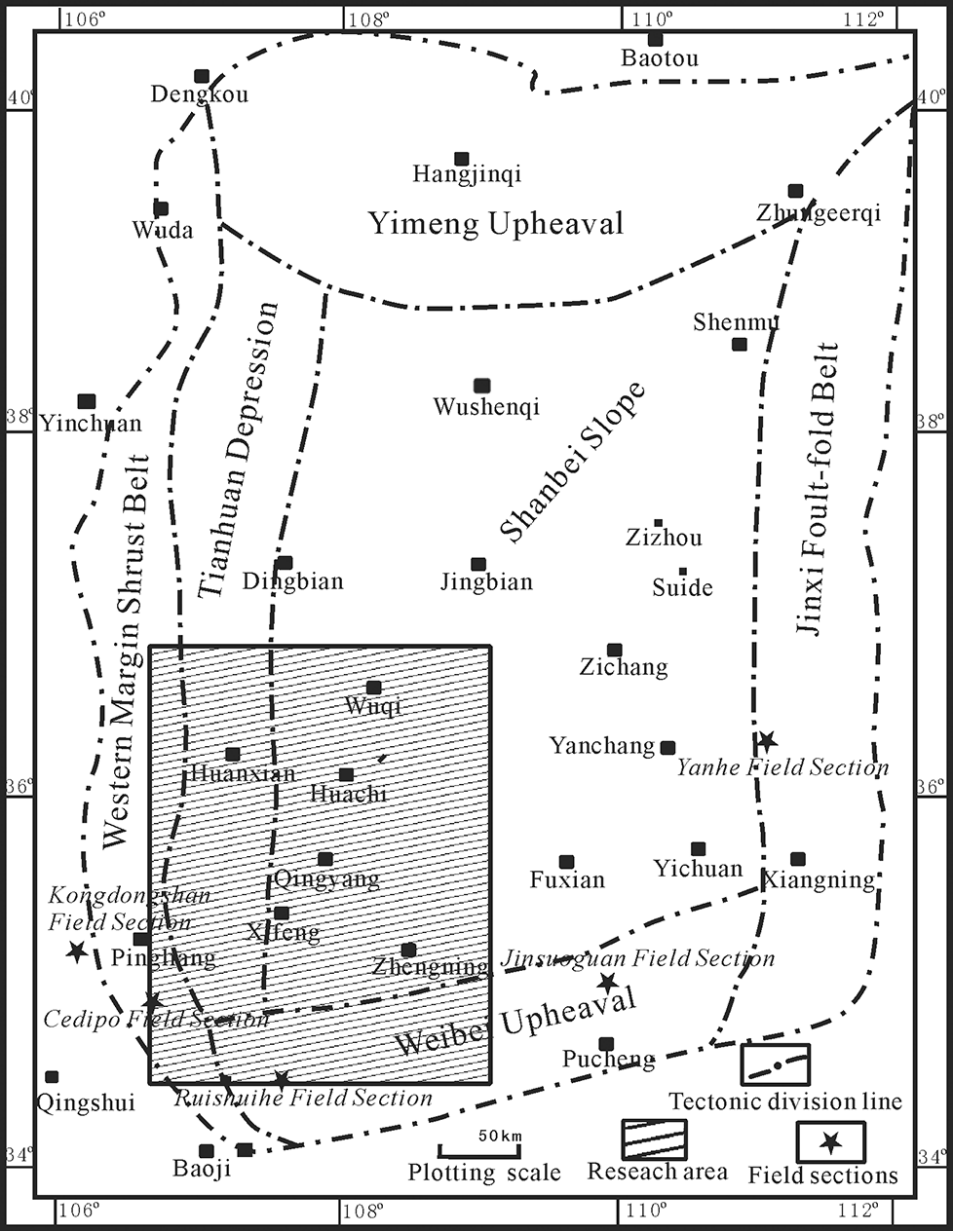
Schematic map of the Ordos Basin showing major tectonic units and the location of the study area.

The Ordos basin belonged to a type of platform structure sedimentary basin. After the Hercynian movement, the Ordos basin became an inland depression basin during a depositional stage following the Late Triassic. During this stage, the feature of inconformity was that of a depocenter and subsidence center, and the center of the sedimentary basin was the same as the center of the depocenter, which had thin and stable deposits. The subsidence center in the western basin was accompanied by thick and varying layers of deposits. The study area was located in the northern middle of the Shanxi slope in the Qingyang nose fold belt, which is a flat structure, and a small nose-like uplift developed along the western monocline (Fig 1). The area is characterized by low elevation in the west and high elevation in the east, with sediments originating from the west and southwest directions. Its system characteristics and the characteristics of the sediment changed with tectonic subsidence and the lake level during the Yangchang period.

## Development Conditions of a Shallow Water Delta

### 3.1 The area remained in a subsidence stage, with wide areas of basin and a slow slope

**Fig 2:**
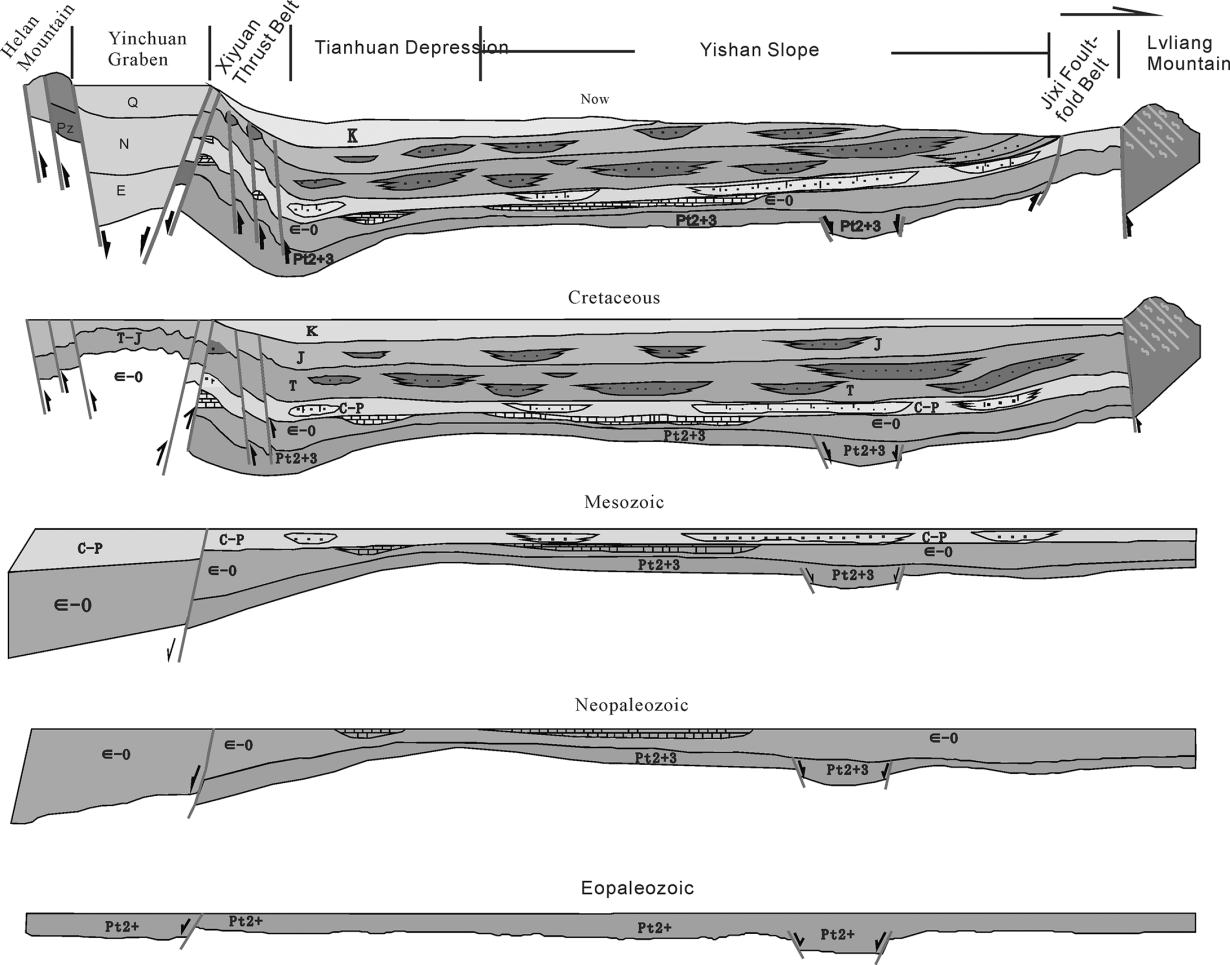
Tectonic-sedimentary evolution of the Ordos basin (red/yellow represent the oil/gas deposits).

The Ordos basin was a craton succession basin[35] that was filled with marine deposits from the early Paleozoic period that turned into sea-land transition deposits in the late Paleozoic (green part), as shown in Fig 2. The ocean terminated by the end of the late Paleozoic and entered the stage of inland lake basin development, and the Yangchang period of the Late Triassic epoch was a prime period in the development of inland lake basins (Fig 2, blue-gray section), as indicated by the deposition of thick, high-quality source rocks[36].

Affected by Indosinian movement, a large and wide area of the water’s surface developed in the Yangchang period of the Late Triassic in the Ordos basin, which was accompanied by the formation of a flat terrain and shallow water caused by wet and dry cyclical climate changes. In addition, the Yimeng uplift, Jinxi flexure belt, Weibei uplift, and thrust belts developed in the western margin and adjacent ancient land. A system of source reservoir cap rocks developed from an adequate supply of terrigenous clastic sediments, such as those of lakes and rivers. The basin formed by enclosing the area until the early Cretaceous, which led to significantly narrowed areas of sedimentary deposits.

There was only one set of shallow lacustrine facies during the process of steady subsidence accumulation of the Yanchang Formation in the Late Triassic for the Ordos basin. The age is equivalent to that of the Carnian to the Rhaetic according to the standard geological age (228.7–199.6 Ma), and the duration was approximately 28 Ma. The entire Yanchang Formation was divided into 10 reservoir groups from bottom to top (from top to bottom, the Chang 1, Chang 2… Chang 10 reservoir group, respectively). The main lithology of the Yanchang Formation was thick massive fine to coarse grain feldspar sandstone. However, the Chang 4 + 5 and Chang 7 formations were mainly composed of mud shale because mud shale was the main hydrocarbon source rock. The mudstone of the Chang 8-Chang 6 formations were rich in plant branch fossil fragments, and leaf segments and flap gill class fossils were also found in the mudstone layer of the Chang 8 formation. Ostracoda as well as scales and fish fossils were found in the mudstone of the Chang 7 and Chang 6 formations. These fossil assemblages reflect the relatively shallow lake sedimentary environment.

### 3.2 Ancient uplift developed in the western and southern marine, with an adequate provenance provided by the humid ancient climate and the strong weathering of parent rock

**Fig 3:**
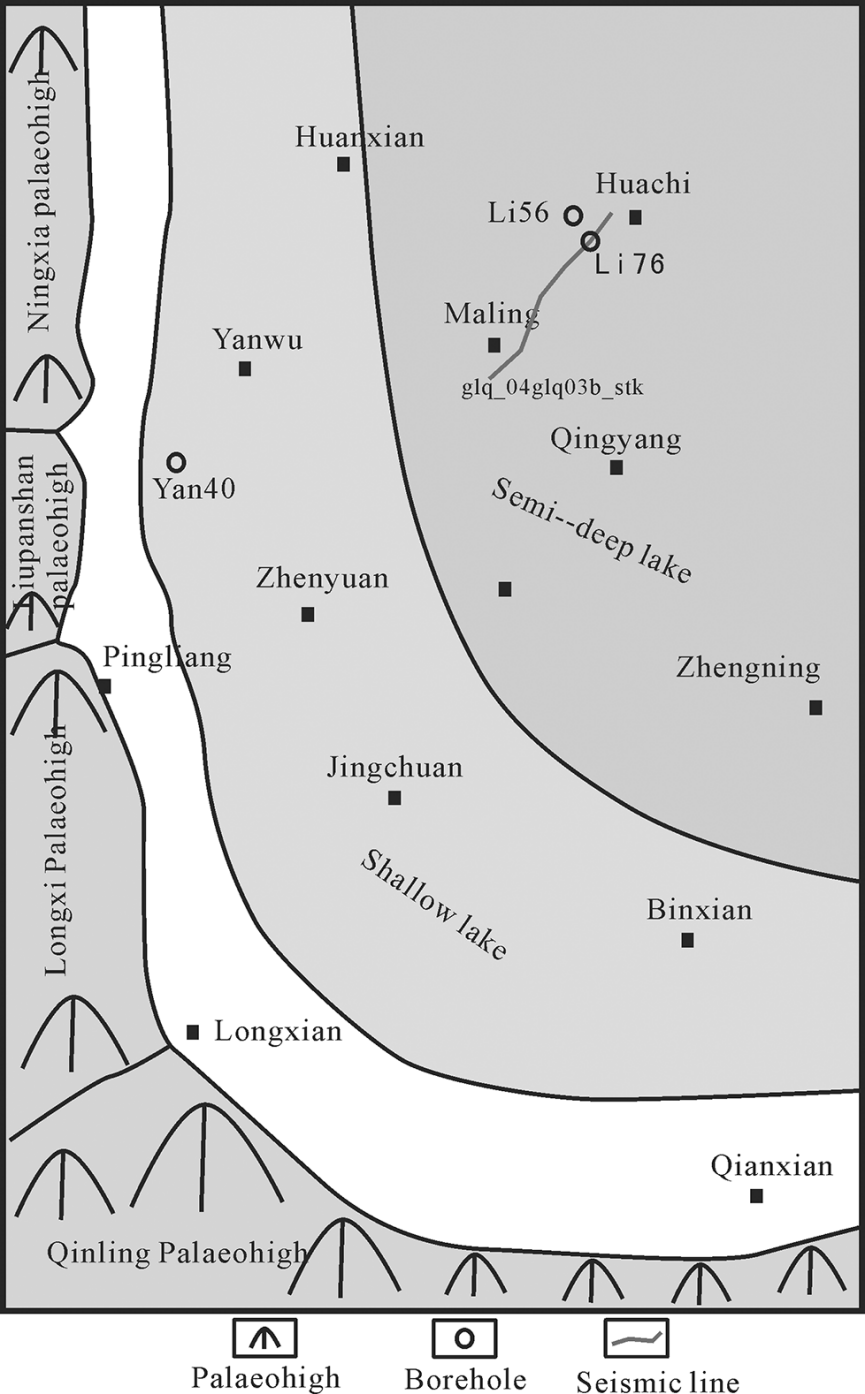
Ancient land in the southwestern margin of the Ordos basin.

There was a nearly NS-trending sinistral shear stress field in eastern China that was caused by the subduction of the Eurasian continent by the Kula Plate in the Mesozoic period, which resulted in an uplift of northern China in a region currently occupied by the Bohai Bay basin. The thrust uplift at the western margin of the Ordos thrust belt began in the Early Triassic and peaked in the Yanshanian period, during which it formed a series of ancient uplifts, including the southern Qinling ancient land, the western margin of the west Gansu ancient land, and the Ningxia Liu Panshan ancient land (Fig 3).

The plants of the Longdong region mainly reflected the tropical and subtropical climatic conditions of evergreen forests in the Middle-Late Triassic. Wet raw ferns flourished with clubmosses and true ferns, such as Cyatheaceae, Dipteridaceae and Selaginellaceae. The flora were mainly distributed in tropical and subtropical moist areas and reflected the wet raw environment. This type of forest vegetation reflected the subtropical climate characteristics of the area. However, the lack of Classopollis of Cheirolepidiaceae and Ephaedripites of Ephedraceae pollen during the Middle-Late Triassic indicated a drought-prone environment. Therefore, the climate of the Longdong areas belonged to a warm-temperate humid subtropical moist climate or hot and humid climate during the Middle-Late Triassic period[37], which was warm and humid and had abundant rainfall. The humid paleoclimate increased the physical weathering of the peripheral ancient land in the Late Triassic period, and it also provided water for the formation of large rivers. Under the alluvial action of large rivers, a significant amount of debris could have provided an adequate provenance for the formation of the shallow water delta.

### 3.3 The lake level cycle changed dramatically due to ancient water turbulence

There was imperceptible horizontal bedding and a continuous rhythm that developed in the deep water areas of the basin. The sedimentary marks of a flysch structure, slot mold and ditch mold indicated deep lake turbidite. The shallow water areas were marked by various types of bedding, including parallel bedding, bedding and sand grain bedding, such as ripple, mix structure and erosion scour. The sediment produced by water was represented by a precipitation-induced impression and featured rill mark level structures.

The Longdong region of the Ordos basin showed distinct cycle variation and frequent changes in lake water level characteristics, based on research related to lithological-electrical analyses, characteristics of the biomarkers, sedimentary structure and lithology marks, such as trace fossils (burrow, footprint, and mark up), and other biological disturbance structure analyses.

## The Sedimentary Features of the Shallow Braided River Delta

### 4.1 Lithology

**Fig 4:**
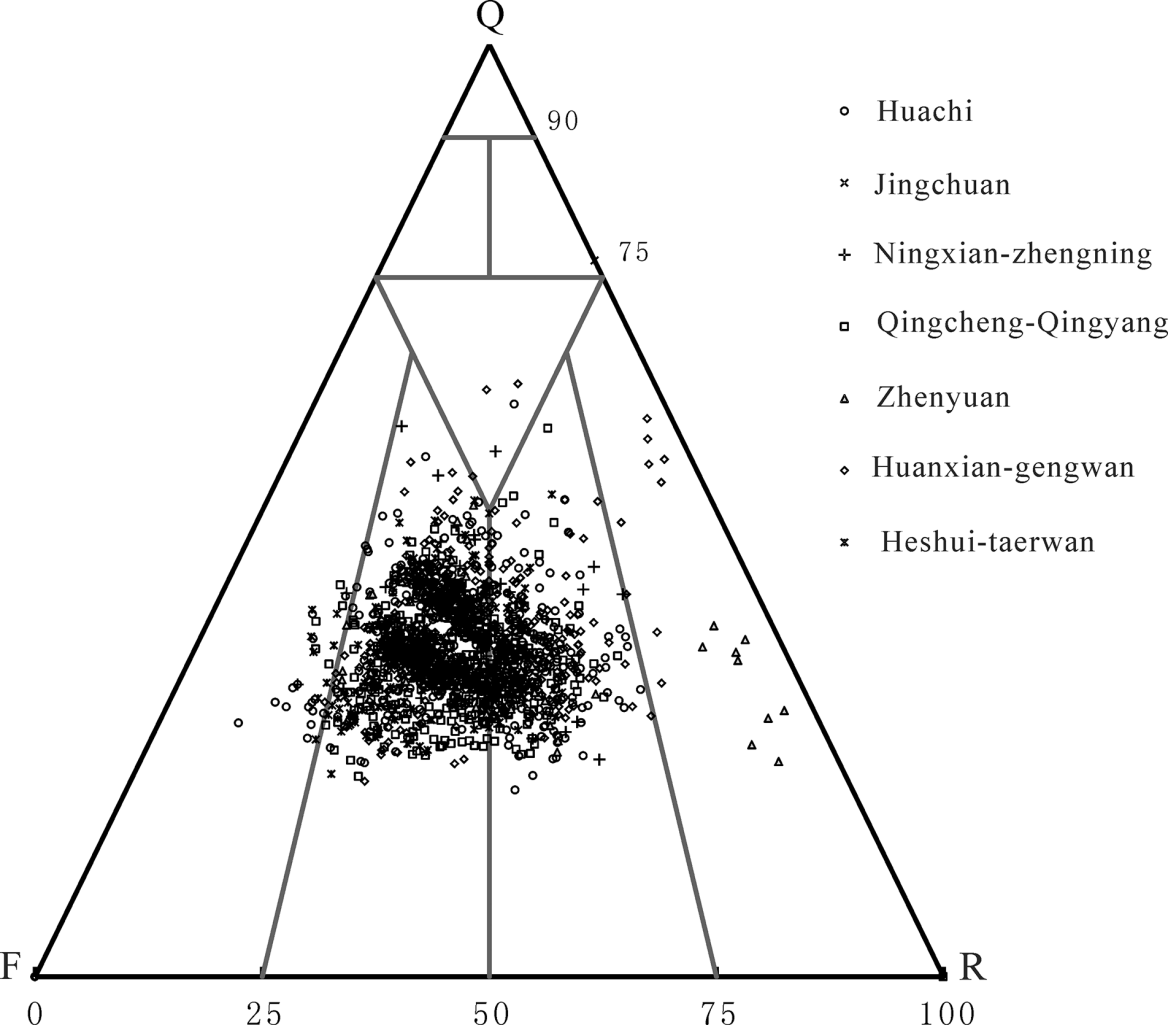
Ternary diagram of the sandstone types of the Yanchang group in the southwestern margin of the Ordos basin.

The Chang 8 and Chang 6 formations were composed of a complex sandstone, as indicated by an outcropped section of the Yanchang Formation in the Ordos basin. This outcrop revealed lithic arkose, feldspathic litharenite and arkose, with a small amount of feldspar lithic quartz sandstone (Fig 4). Each component was contained in the sandstone, including quartz (average 32.03%), feldspar (average 31.05%), and a high lithic content (average 36.30%). However, the type of rock debris varied. The Kongdong Mountain section mainly contains magmatic rock debris (average 10.60%) and sedimentary rock debris (average 22.00%), and the Cedipo and Ruishui River profile section mainly features magmatic rock and metamorphic rock debris. Compared to the southwest margin of the basin outcrop section, the Yanhe profile section in the eastern part of the basin showed a single type of sandstone and mineral composition, mainly feldspar sandstone and lithic feldspar sandstone. Each component was contained in the sandstone, including quartz and feldspar, with higher quartz (average 31.50%) and feldspar (41.65%) contents and a lower lithic content (average 6.8%). A large amount of debris consisted of metamorphic rock and sedimentary rock, and a small portion of magmatic rock was observed.

**Fig 5:**
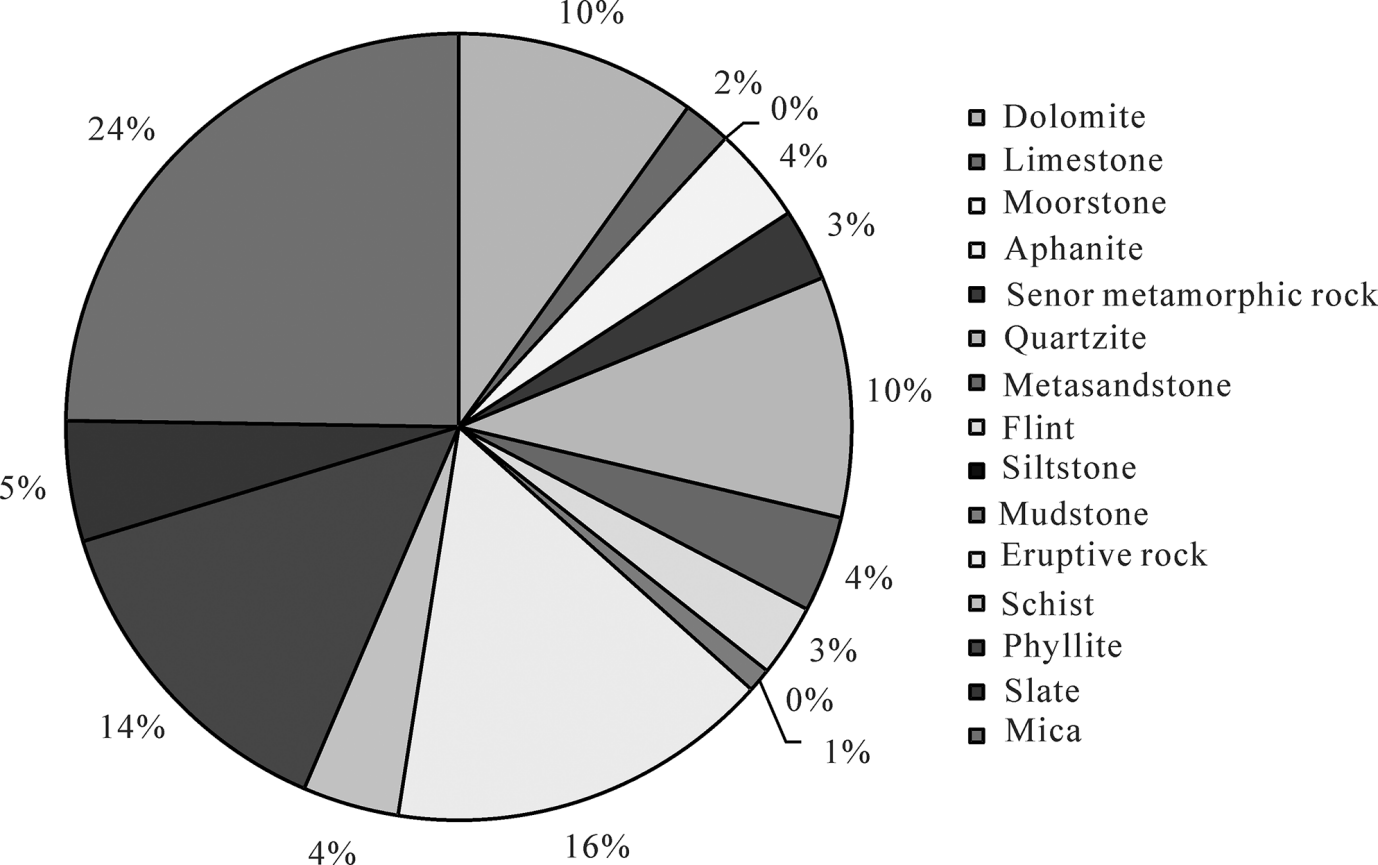
Debris composition of the Yanchang Fm. in the southwestern margin of the Ordos basin.

Analysis of rigid and plastic mica and lithic samples from the Yanchang Formation revealed that debris from the reservoirs was mainly composed of plastic debris, accounting for approximately 64% of the total debris and mica. Among the debris, mica, eruptive rock debris and phyllite rock debris were relatively well developed, and each accounted for approximately 24%, 16% and 24% of the total composition, respectively. The rigid lithic content was observed to be low, constituting approximately 36% of the total debris and mica content. Dolomite debris and quartzite debris were relatively well developed and accounted for approximately 10% of the total composition. The composition maturity was low, with a mean of 0.65 (Fig 5). The composition maturity of the stratum below the Chang 7 group in the Yanchang Formation was less than 0.5, whereas that of the upper stratum was relatively high and even reached values greater than 0.5[25].

Based on the aforementioned data, the Yanchang Formation of the Longdong region showed a sedimentary environment exhibiting strong water performance and was close to the provenance. This result is reflected by the relatively low quartz content, high feldspar and lithic content and lower compositional maturity of the sandstone. The plastic components in the debris content were relatively high, and the rigid component content was low; in addition, the content of the matrix was low. The primary rock types were coarse- to fine-grained lithic arkose and feldspathic lithic sandstone with medium separation, and the separation of the Chang 6 and 8 groups in the Yanchang Formation was relatively poor, with most of the particles having a subangular shape.

### 4.2 Sedimentary structure characteristics

The southwest margin of the Ordos basin possesses typical strong hydrodynamic depositional features, as evidenced by the distinct size of the erosion interface, the directional arrangement of gravel, trough cross-bedding, wedge cross-bedding, parallel bedding, etc.

**Fig 6:**
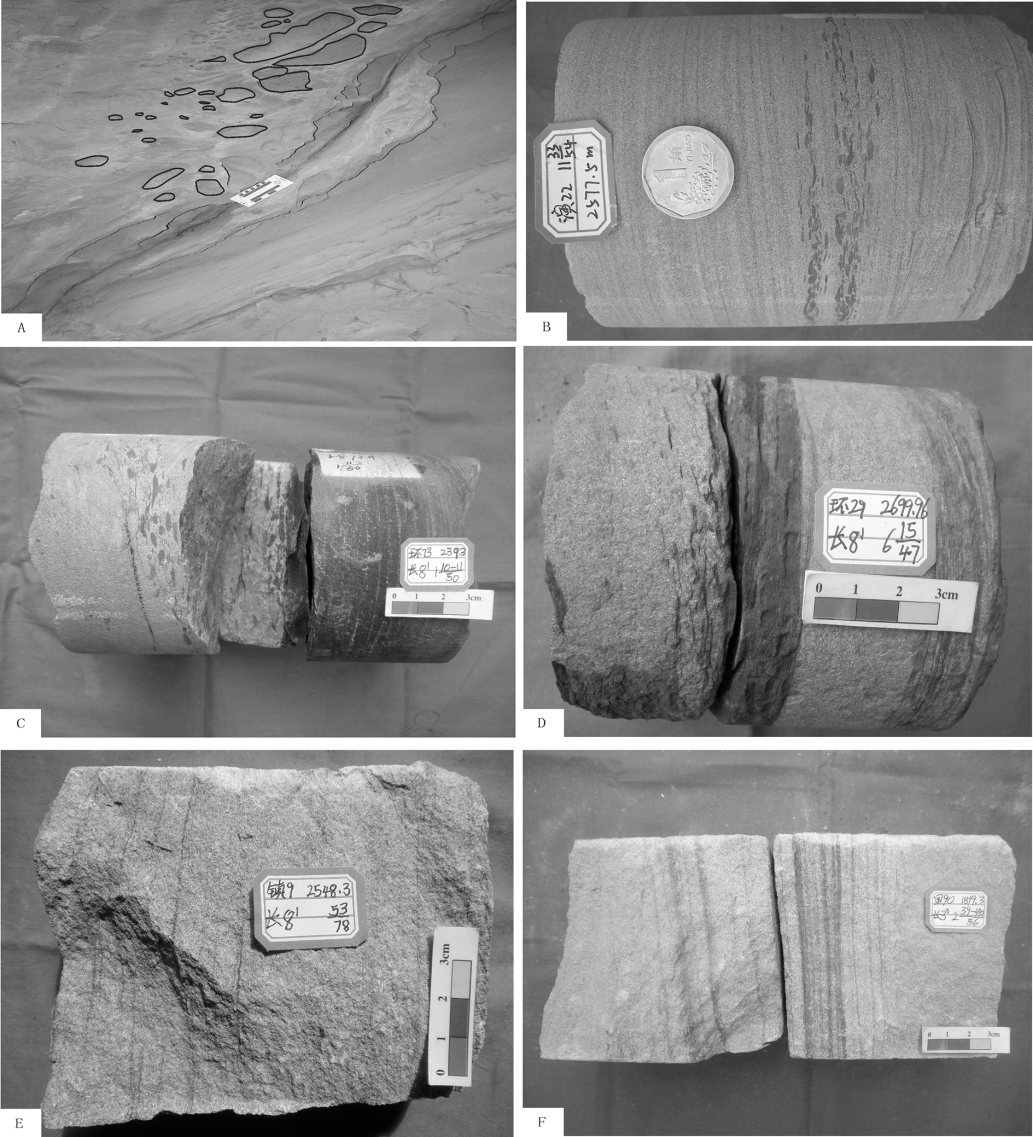
Typical sedimentary structure of the shallow water delta of the Yanchang Fm. in the southwestern margin of the Ordos Basin. A: Ruihe profile section, including the thick sandstone located at the bottom of the Chang 8 formation and gravel directional arrangement; B: Well Yan 22, TVD2577.5 m, Chang 6 formation, directional distribution of mud stone and gravel layers; C: Well Huan 73, TVD2392 m, Chang 8–1 formation, wash surface structure and sandstone gravel at the bottom in a directional arrangement; D: Well Huan 27, TVD269 9.96 m, Chang 8–1 formation, scour surface at the bottom of the sandstone; E: Well Zhen 9, TVD 2548.3 m, Chang 8–1 formation, large wedge cross-bedding; and F: Well Li 92, TVD 1819.3 m, Chang 3–3 formation, wedge cross-bedding and parallel bedding.

The Ruishui River outcrop section is a representative section of the “slender Yanchang Formation" in the southwest margin of the Ordos basin. The Chang 10 group of the Yanchang Formation in this section developed the following characteristics: a thick layer of gray, celadon or massive fine sandstone (40 ~ 100 m), large tabular cross-bedding, and wedge cross-bedding. The lower formation developed amaranth mudstone unconformably in contact with the Zhifang Fm., and a wormhole was also visible. A distinct fault developed between the interface layer, but there was no obvious dislocation. The Chang 9 and Chang 8 reservoirs occurred mainly on dark gray to gray thick massive sandstone that was clipped with a thin carbonaceous mudstone layer (20 ~ 30 cm); in addition, large tabular cross-bedding and scour mud gravel could be observed (Fig 6A). A directional arrangement of gravel, which reached a thickness of 10 cm among the sandstone gravel, had an average particle size of 3~5 cm. Another carbonaceous mudstone thin layer (20 to 30 cm) was associated with the delta channel sandstone thick layer. All of these characteristics reflect a shallow sedimentary environment.

All types of features associated with the ancient environment can be commonly found in cores of the Yanchang Formation in the Longdong area, including a wash surface (Fig 6B, 6C and 6D), tabular cross-bedding (Fig 6E), wedge cross-bedding (Fig 6F), trough cross-bedding and parallel bedding. These features mainly appear in the thick sandstone of the distributary channel. Multiple cross-beddings with laminated flat, tilt progradations, and an oblique orientation with formation interfaces and formation thicknesses larger than 3 cm all reflect strong current and shallow sedimentary characteristics.

### 4.3 Sand body vertical superimposed development features

The branch channel was well developed in the shallow water delta plain and front portion of the Yanchang Formation. Because of the shallow water, the flat bottom of the river, the sufficient source supply, and frequent diversions, a continuous positive rhythm occurred from the direction of the lake basin edge to the center of the lake in the delta plain and front sedimentary environments, which was accompanied by an increased thickening intercalation. A "without mud" intermittent positive rhythm developed in the delta plain, and a “within mud" intermittent positive rhythm mainly developed in the delta front[25].

**Fig 7:**
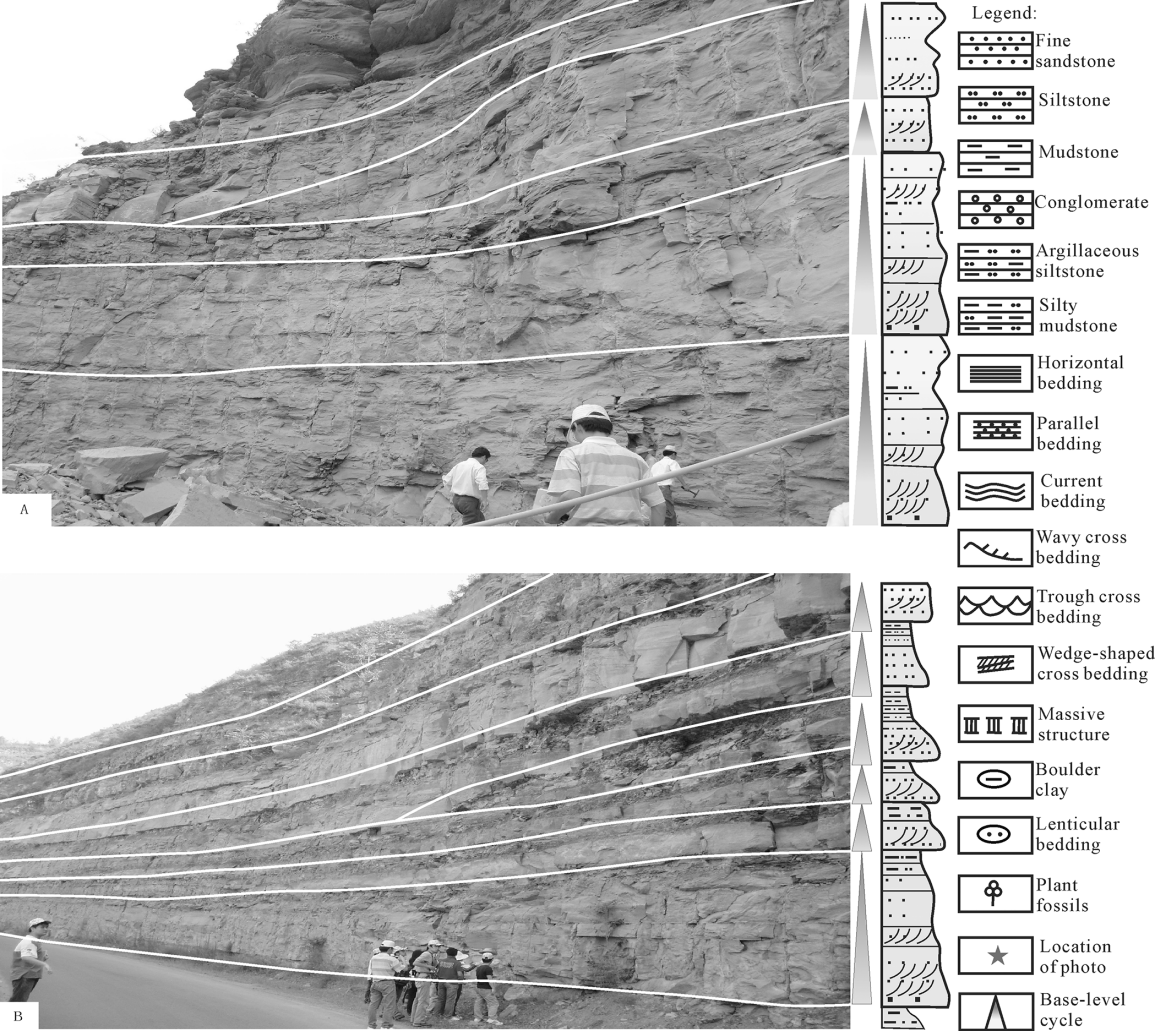
Detected outcrop profiles illustrating the composition of the submarine distributary sand bodies in the Yanhe section.

**Fig 8:**
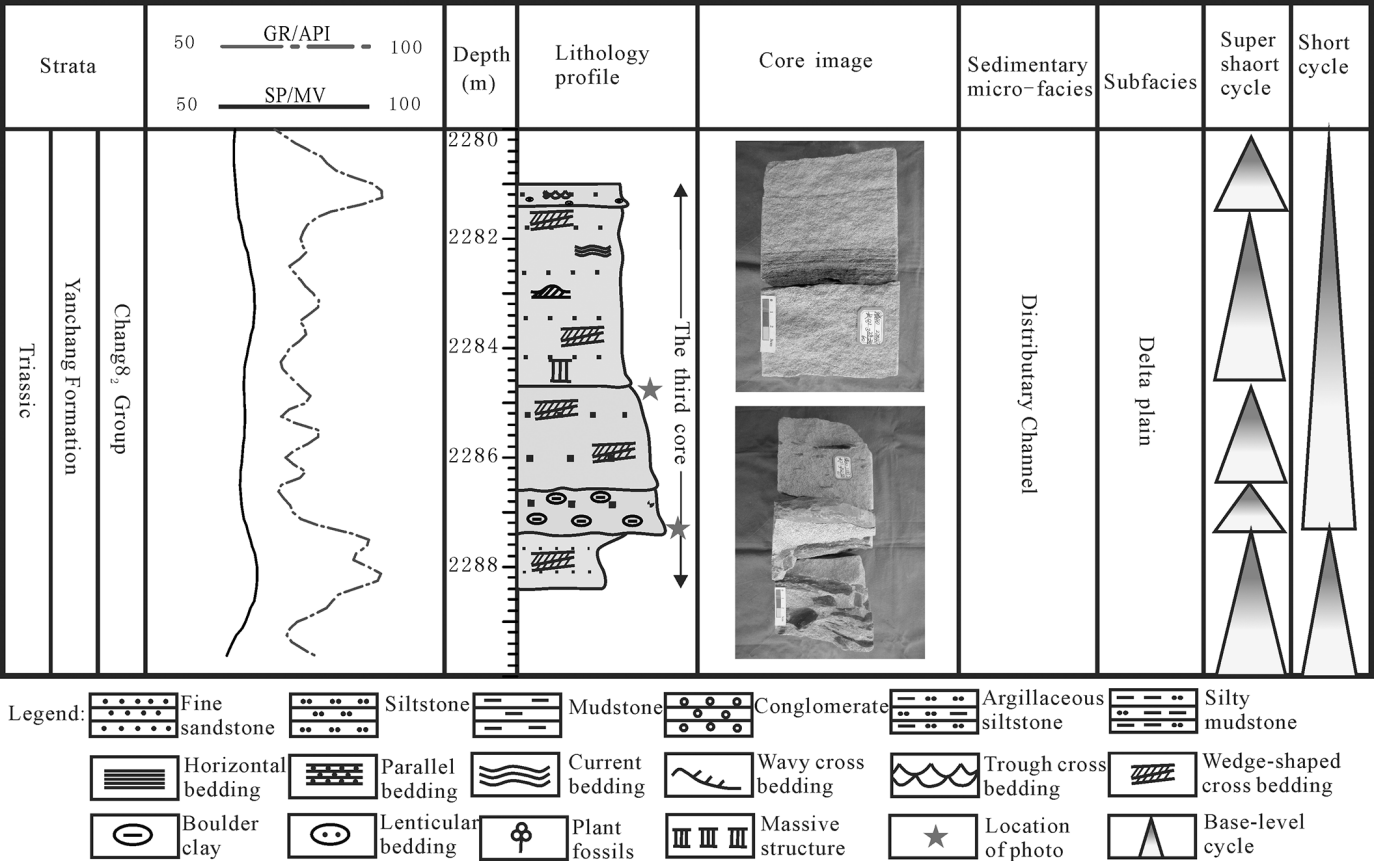
Sedimentary cycles of the shallow braided delta of the Yanchang Fm. in the Longdong area of the Ordos basin (Wll Yan40).

The "without mud" intermittent positive rhythm refers to no mudstone (clip) existing between the two positive rhythm insulation layers, constitutes the discontinuity of the sedimentary lithology of the scour contact between sand and reflects frequent and strong hydrodynamic activities. A washing surface existed in the thick sand body, and a gravel directional arrangement and wedge cross-bedding was developed in the thick sandstone. The "without mud" intermittent positive rhythm of the sedimentary sequence was mainly developed in the delta plains and underwater branch channels in the delta front, corresponding to a lithology composed of gravelly coarse sandstones. Large cross-bedding developed, and a sandstone thickness in a single layer measuring approximately 0.5–3 m could be found in the Chang 3 group of the Yanhe profile section (Fig 7A) and the Well Yan 40 (Fig 8).

The “within mud" intermittent positive rhythm indicates the existence of mudstone (clip) between the two positive rhythm insulation layers, and it reflects the strong hydrodynamic effect of the process of subaqueous distributary channel sedimentation. The rhythm mainly developed in the subaqueous distributary channel in the delta front. Medium-sized cross-bedding was developed, and the main lithology was fine sandstone; the single-layer thickness of sandstone was 1.5–3.1 m. A series of environmental indicators was observed, including a wash surface, directional arrangement of sandstone gravel, wedge cross-bedding in fine sandstone or wavy parallel bedding, cross-bedding among fine sandstone, silty mudstone or argillaceous siltstone with horizontal bedding, bottom rock interface contact with underlying sandstone at the wash surface, and upper sandstone contact with the overlying sandstone or mudstone. These features could be found in Well Li 56 (Fig 7B) and the Chang 8–1 group of the Yanhe profile section (Fig 7B).

### 4.4 Plane distribution characteristics of a sand body

The provenance of the Yanchang Formation in the Longdong region is derived from the southwest shallow braided delta of the Ordos basin. The delta front is a composite of 3–7 underwater branch channels, each of which could be split into 2–4 secondary branch rivers. Underwater branch channels presented branching and were mixed in the distribution. The single channel sandstone had a thickness of 0.8–6.6 m, the cumulative thickness of the channel sandstone was approximately 4–65 m, and the sand stratum ratio was 5–90%.

**Fig 9:**
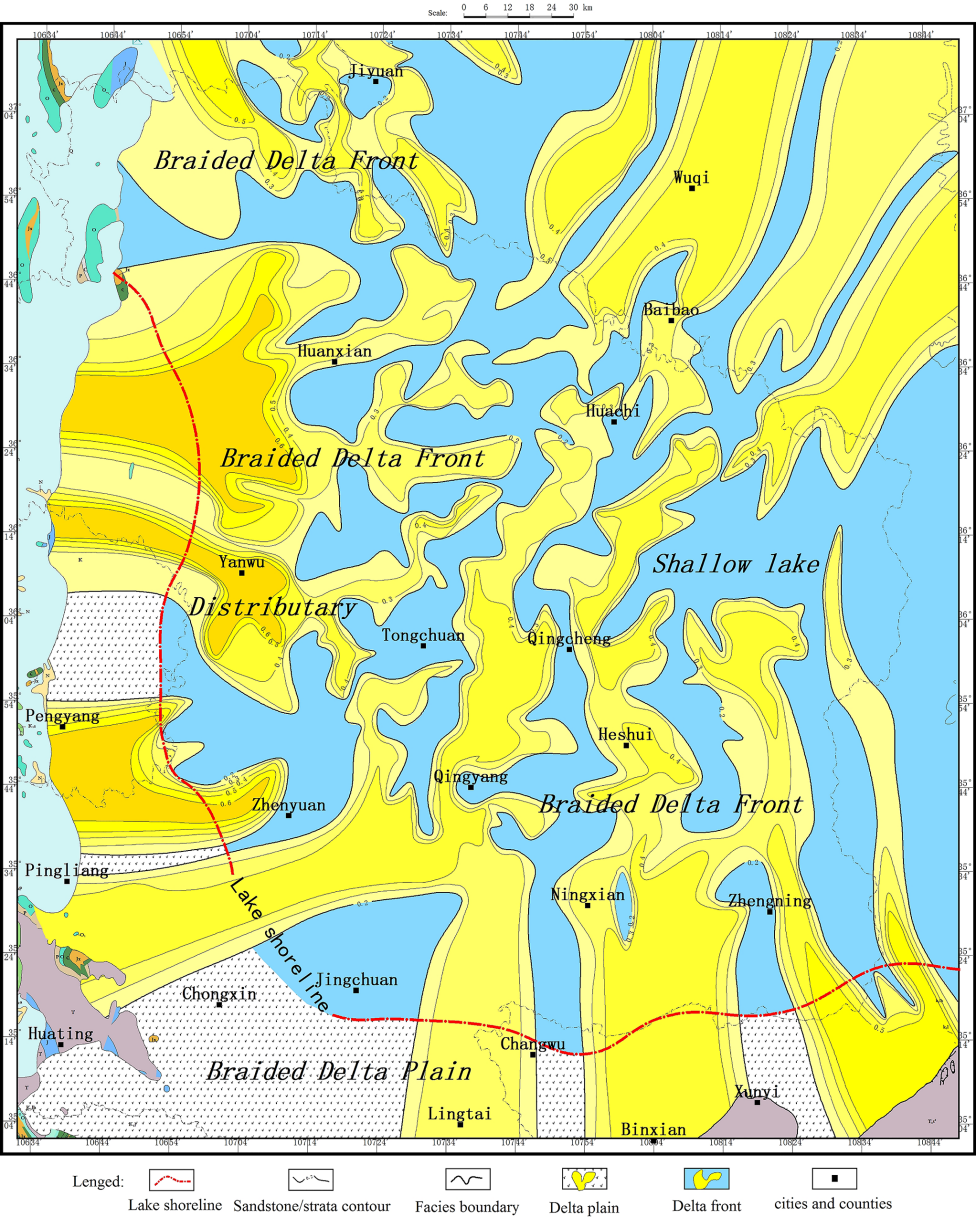
Depositional system of the Yanchang Fm. (8^1^) in the Longdong area of the Ordos basin.

Single branches could extend over long distances in the direction of the central of basin. During the basin’s peak stage of evolution, the lake level increased and the lake surface area was large. The distance from the delta front to the center of the basin was relatively short at 9–46 km. During the evolution of the basin, a no-peak stage occurred and the lake level declined. The area of the lake was relatively small and had a shallow water level, and the distance from the delta front to the center of the basin was relatively long at 18–100 km (Fig 9). In short, the Yanchang Formation had the underwater distributary channel sedimentary characteristics of a delta front in a shallow water environment, which was revealed by an underwater distributary channel with a thick sand body that extended over long distances; this finding suggests the presence of adequate supplies, shallow water, and strong hydrodynamic forces.

### 4.5 The seismic response characteristics

**Fig 10:**
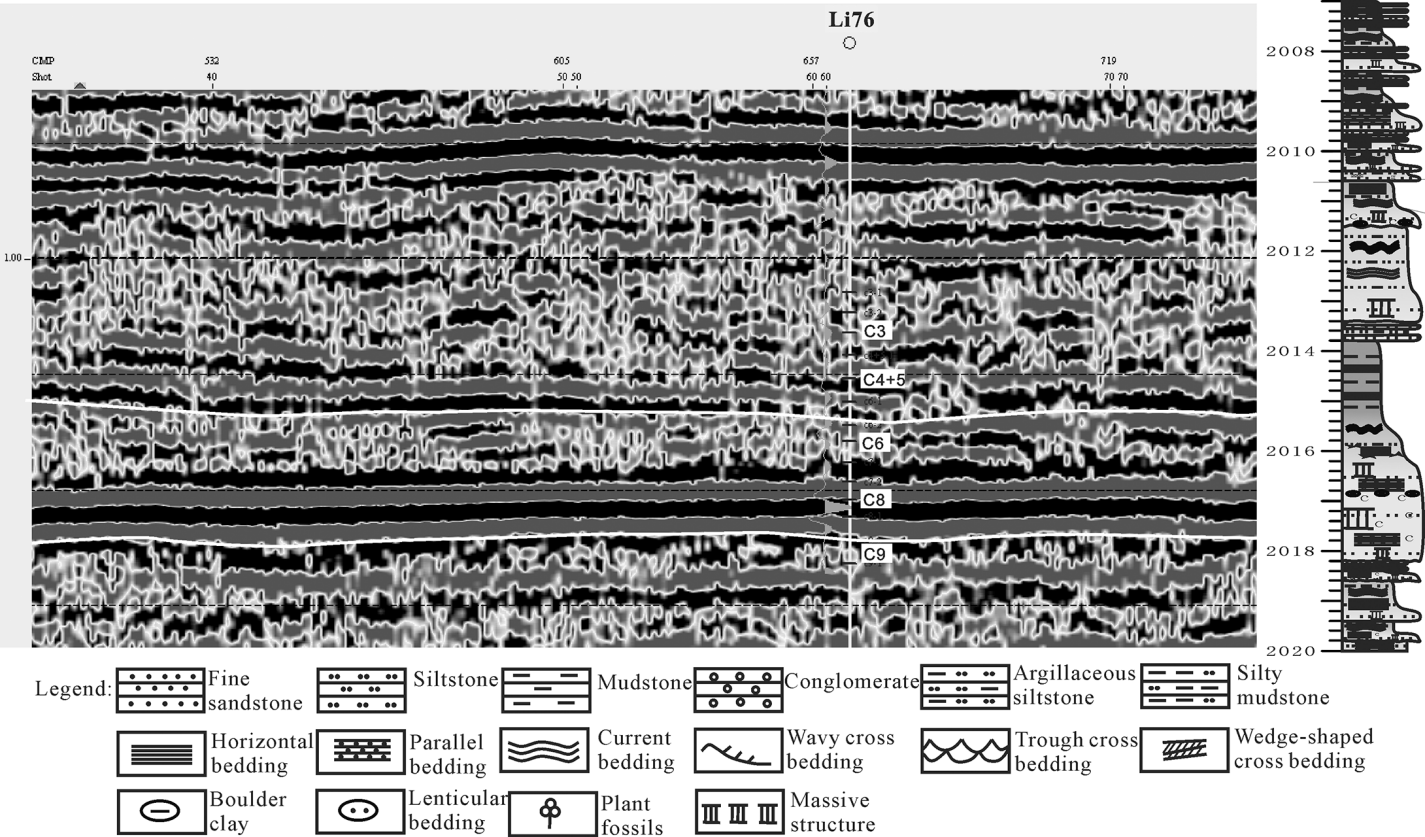
Seismic reflection of the shallow braided delta of the Yanchang Fm. in the Longdong area (line:glq_04glq03b_stk).

During the Yanchang Formation sedimentary period, the Ordos basin was in the depression stage, accompanied by a shallow water level, a large amount of water space and adequate supplies. In addition, there were frequent fluctuations in the lake level; therefore, a braided delta with coarse grains developed under strong hydrodynamic effects in the Longdong region. Several types of seismic progradation reflections indicated the delta sediment transfer direction, which pointed to the center of the basin. The foreset form represented the delta sedimentary types, and the foreset shape indicated the sedimentary environment with hydrodynamic effects as well as a terrain slope that was steep or slow. The study area’s progradation of seismic reflection appeared relatively flat and typically ranged from 2 to 4 in the phase axis, with a strong seismic reflection amplitude. In addition, the reflection showed better continuity with a low angle and extended over long distances (up to 50 km). The slope progradation seismic reflection indicated a braided delta sedimentary environment with a shallow water level and strong hydrodynamic forces. It could be concluded from the seismic profile (Fig 10) that the seismic response of the breadth of the braided river delta could reach 3–5 km, which was composed of one lenticular compound and domal structures that were reflected in the seismic response. All of the seismic reflections could be found in Well 76 (TVD2007.1–2020.0 m) of the Chang 6 group of the Yanchang Formation by drilling. A typical delta front underwater branch river with intermittent positive rhythm was observed in the sedimentary sequence; from bottom to top, the sequence transitioned from sandstone or mudstone with a boulder wash surface, to medium-sized fine sandstone with wedge cross-bedding, to sandstone with visible parallel bedding. The thickness of the intermittent positive rhythm was approximately 0.5–5.6 m.

## Sedimentary Model of the Shallow Braided Delta

The braided delta had a "small plain, big front” distribution and the vertical evolution characteristics of the Yanchang Formation in the Longdong region of the Ordos basin[25]. The delta’s sedimentary evolution was clearly controlled by tectonic movement and lake level changes. Due to the distinct lake level changes during the period of lake water levels, branch channels extended to the central basin; however, the branch channels were reformed by the increased lake levels, which resulted in a braided delta with "small plain, big front” sedimentary characteristics[25].

**Fig 11:**
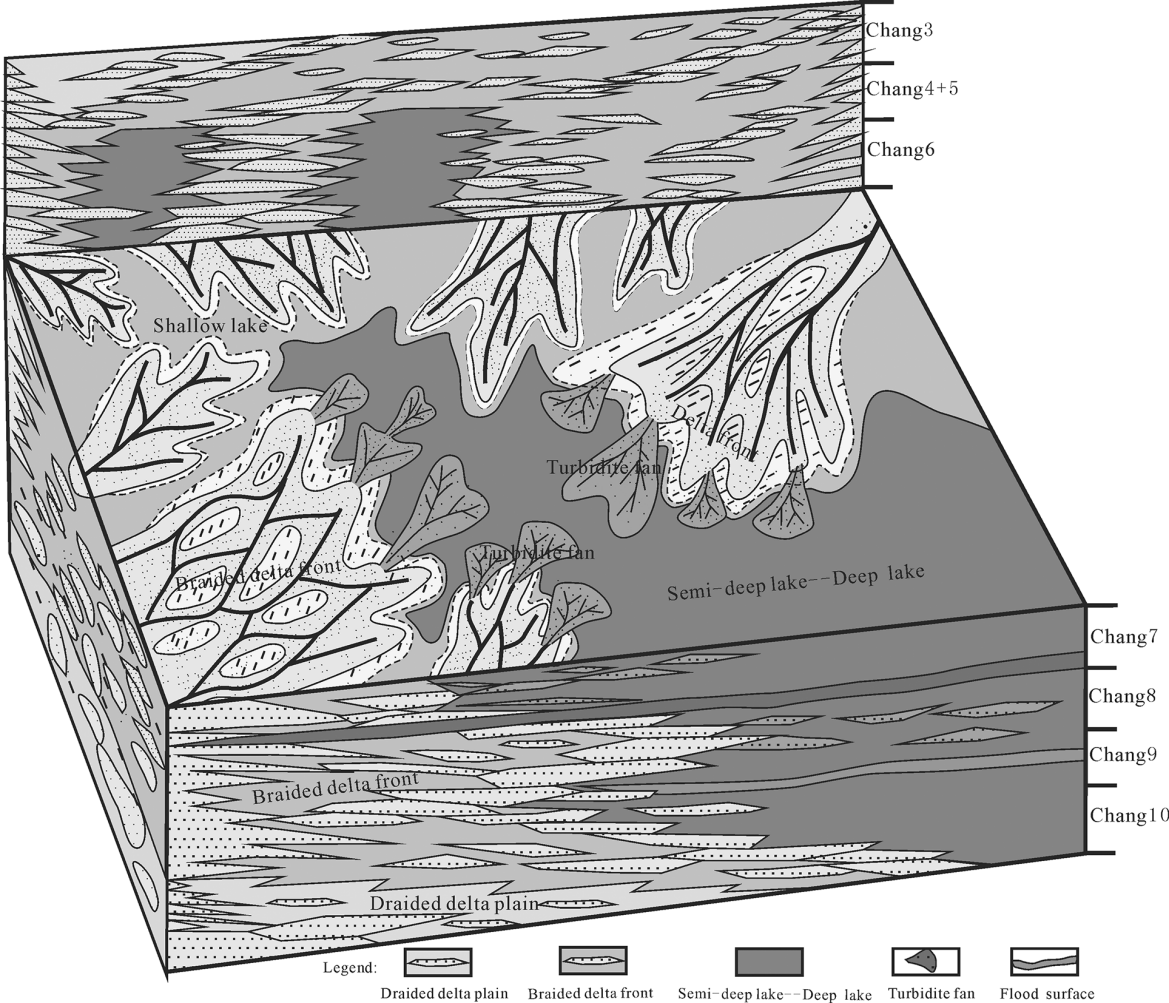
Schematic sedimentary model showing the evolution of the Yanchang Formation in the southwestern margin of the Ordos basin.

The Yanchang Formation of the Ordos basin formed in a complete cycle of the inland depression lake basin, which experienced 4 complete return stages from the initial depression stage as well as a severe depression stage to an uplift shrinking vanished stage[35]. The sedimentary system scale, sedimentary characteristics and evolution process of the shallow water braided delta were controlled by tectonic evolution (Fig 11).

During the Chang 10-Chang 9 sedimentary period, the Ordos basin was under continuous stress, which was inherited from the Early Triassic to Middle Triassic. The Ordos basin experienced an initial stage characterized by a lake basin depression; then, the basin began to develop, and the initial shape of lakes formed within shallow water levels. The study area is close to a provenance[38]. Plenty of detrital material was deposited in the local area[33,39], and a shallow lake sedimentary system developed in the study area. Four braided river delta fronts were distributed from the northwest to southeast on the plain. The width of the braided river delta front was approximately 40 km in the southwestern part. The formation thickness was 31–90 m, the sand body thickness was 8–28 m, and the sand formation ratio was approximately 20–50%; however, the local area was composed almost entirely of sandstone (Yan 47, the ratio could reach up to 100%).

**Fig 12:**
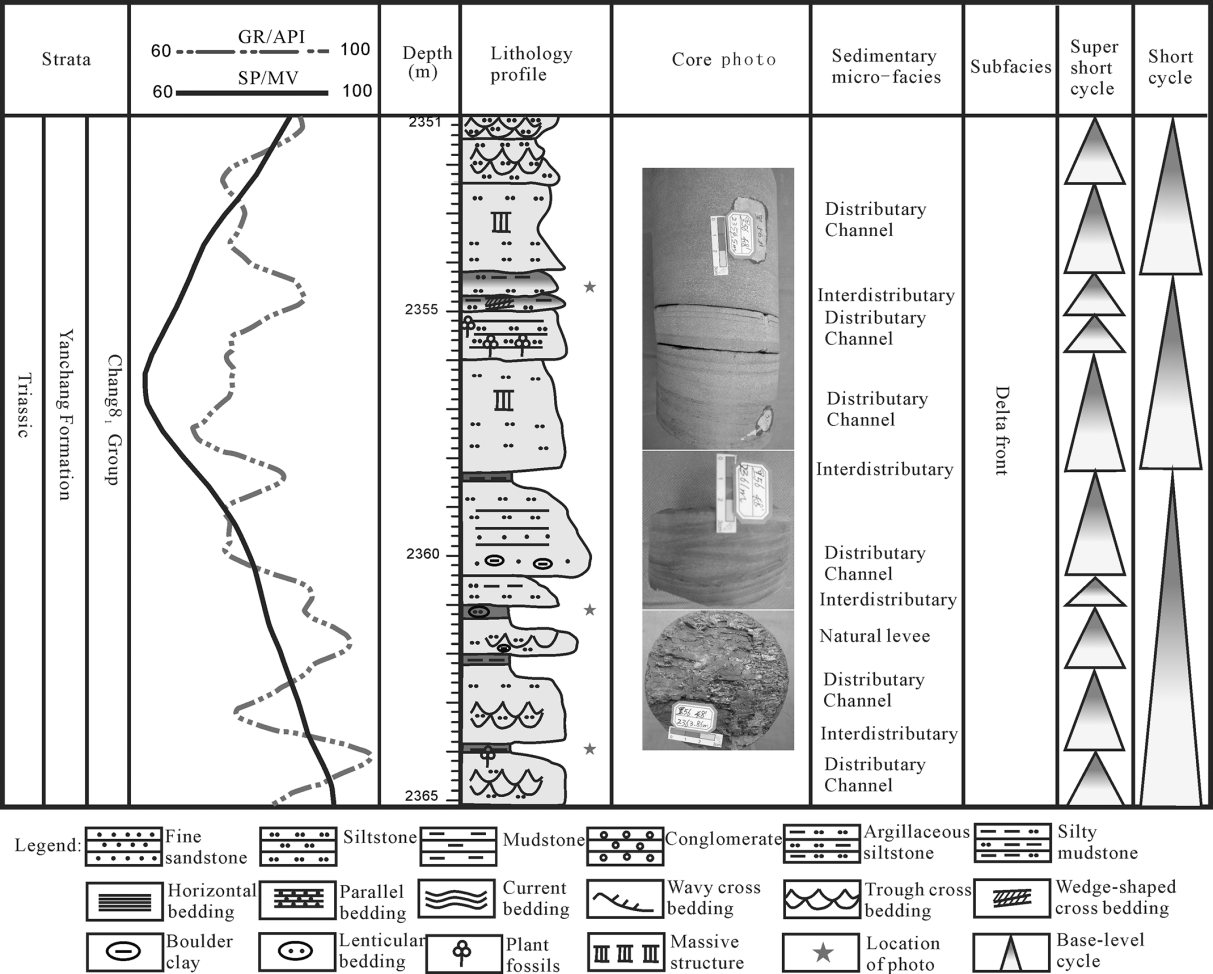
Sedimentary cycles of the shallow braided delta of the Yanchang Fm. in the Longdong area of the Ordos basin (Wll Li56).

During the Chang 8 sedimentary period, the lake basin had a widened scope and large water depth, and it was characterized by a large area of lakeside swamping that was associated with the braided river delta channel sand body and a thin coal layer. The braided delta plain was mainly composed of coarse sandstone gravel, sandstone gravel and peat swamp deposits. The plain distributary channel deposit was mostly typical, with multiple superpositions at the bottom of the scour surface, developmental groove cross-bedding, wedge cross-bedding and parallel bedding. Overlying the thin layer (less than 1 m) of a coal seam was the sedimentation of the vertical structure of the discontinuous depositional sequence (Fig 12).

The braided delta front mainly developed in alternating layers of fine sandstone and argillaceous siltstone and was characterized by a common scour surface, parallel bedding, trough cross-bedding, wavy cross-bedding, slump deformation structure, structure and plant debris bones wormholes. In the vertical direction, sedimentation particles of up to a fine size were in contact with the underlying dark mudstone mutation of the sedimentary sequence, especially in the long 8 reservoir group of the common coal core wire (Fig 12).

During the Chang 7 sedimentary period, the basin water level sharply increased and the basin area expanded significantly when a set of large thickness dark mudstones with high organic and shale contents was developed (Zhang Jiatan shale); in addition, the delta receded to the southwest provenance, and the delta area receded less than 40 km in the study area.

During the Chang 6 to Chang 4+5 sedimentary period, the lake basin returned to the uplift stage and began to shrink. This process was associated with a gradually increasing supply, and a series of constructive river-lake delta sedimentary sand was developed in the basin. The Wu Qi delta extended from the northeast to the Baibao region, and a set of thick sand layers was deposited in the meandering river delta front.

During the Chang 3 to Chang 1 sedimentary period, the lake basin entered the shrinking to dying stage. Tectonic activity decreased significantly, and the lake basin filled with ooze and then atrophied and died gradually. In addition, during the Chang 3 sedimentary period, a significant deltaic progradation occurred on the margin of the basin, and the delta immigrated from the southwest to the lake center. During the Chang 2–1 sedimentary period, due to the strong denudation followed by the huge uplift of the basin, the southwest margin of the basin was nearly denuded, and the northwest section of the reservoir remained incomplete. A significant portion of the basin was swamped, and a coal seam and wide coal line developed. The entire lake basin was submerged and buried.

## Conclusions

During the Yanchang Formation period in the late Triassic, a shallow braided river delta developed under favorable geological conditions, i.e., shallow water and a large-area basin with a flat terrain and wide and shallow water areas. In addition, there was a wet and dry cyclical climate, ancient water turbulence, dramatic changes in the depth cycle, ancient uplift development, strong weathering on the parent rock, and abundant supply.

An underwater distributary channel and mouth bar developed in the shallow braided river delta but were not well developed in the Yanchang Formation. The characteristics of these structures were indicative of a thin coal seam line (coal). All of the abovementioned features indicated a shallow water braided river delta with grain sediment granularity, plastic debris, and sediment of mature composition and structure that reflected the strong hydrodynamic environment of large tabular cross-bedding, wedge cross-bedding, multiple positive rhythms superimposed to form a thick layer of sand, and procreation of seismic reflections that appeared to be relatively flat and low-angle.

During the development of the shallow braided river delta in the Yanchang Formation sedimentary period, the sedimentary center was close to a provenance, the width of the river was even greater, a shallow sedimentary structure and a sedimentary rhythm developed, and delta development was mainly controlled by tectonic activity and changes in lake levels. As a result, a "small plain, big front" characteristic was eventually developed in the shallow braided river delta sedimentary system.
